# The impact of excess liver copper concentrations on response to a bovine respiratory disease challenge in lightweight beef-on-dairy crossbred steers

**DOI:** 10.1093/jas/skaf218

**Published:** 2025-07-09

**Authors:** Jacob A Henderson, Olivia N Genther-Schroeder, Stephanie L Hansen, Jodi L McGill

**Affiliations:** Department of Animal Science, Iowa State University, Ames, IA 50011; Land O’ Lakes, Inc., Gray Summit, MO 63039; Department of Animal Science, Iowa State University, Ames, IA 50011; Department of Veterinary Microbiology and Preventative Medicine, Iowa State University, Ames, IA 50011

**Keywords:** beef-on-dairy, bovine respiratory disease, copper, immune function

## Abstract

Beef-on-dairy crossbred steers are exposed to greater amounts of copper (**Cu**), which may impact their resiliency to disease. To test this, 26 weaned beef-on-dairy steers (95.2 ± 7.2 kg; ~8 wk old) were blocked by weight to pens, and pens were randomly assigned to two target liver Cu statuses: adequate (**ADE**) and HIGH. To achieve target statuses, ADE and HIGH were fed diets containing no supplemental Cu and 20 mg Cu/kg diet DM, respectively, for 120 d before enrollment in a 13-d bovine respiratory disease challenge. Liver Cu prior to challenge averaged 279 and 608 mg Cu/kg liver DM for ADE and HIGH, respectively. Steers were infected with 10^4^TCID_50_ BRSV on day 0 via aerosol inoculation. On day 5 postinfection, steers were intratracheally infected with 5 × 10^8^ CFU *Mannheimia haemolytica*. A trained observer scored steers for depression, appetite, and respiration from days 0 to 14. On days 0, 5, 7, 10, and 13, thoracic ultrasound was used to score animals based on the degree of lung consolidation and lesions, and jugular blood was collected. Categorical variables (clinical and lung scores) and continuous variables were analyzed using PROC GLIMMIX and PROC MIXED of SAS 9.4 (Cary, NC), respectively. Clinical scores were affected by treatment × day (*P* = 0.04), where HIGH experienced a sharper increase in clinical scores in response to disease compared to ADE (*P* < 0.01) and remained higher throughout the remainder of the disease challenge (0.01 < *P* ≤ 0.08). Over the entire challenge, HIGH steers tended to have greater lung consolidation than ADE (*P* = 0.08). While no differences in haptoglobin were detected between treatments (*P* = 0.96), both treatments experienced marked increases in haptoglobin on day 7 postinfection (*P *< 0.01), indicating inflammation in response to disease. There was a tendency for a treatment × day interaction (*P* = 0.07) for plasma Cu, where HIGH steers exhibited less dramatic increases in plasma Cu than ADE. Plasma Zn was affected by treatment × day (*P *< 0.01) where HIGH steers did not change over time and ADE exhibited decreased plasma Zn in response to disease, characteristic of a classical nutritional immunity response. Ferric reducing antioxidant power did not differ by treatment (*P *= 0.33); however, both treatments decreased on day 7 postinfection (*P* < 0.01), indicating increased antioxidant demands. In conclusion, these results suggest excessive liver Cu concentrations result in greater disease severity and immune dysfunction in beef-on-dairy steers.

## Teaser

Beef-on-dairy crossbred calves commonly enter the feedlot with greater liver Cu concentrations than calves raised in conventional beef production systems, which may contribute to inflammation and immune dysfunction. In this experiment, steers with excessive liver Cu concentrations displayed heightened severity of disease, increased mucus buildup in the lungs, and an impaired nutritional immunity response to infection.

## Introduction

Beef-on-dairy crossbred cattle have become an increasingly critical component of the U.S. beef industry, as beef semen sales in 2020 were nearly 200% greater than the previous 5-yr average (NAAB, 2021); the majority of this increase is attributable to use in beef-on-dairy crossbreeding (Foraker et al., 2022). Bovine respiratory disease (**BRD**) is one of the most common and costly diseases in the beef industry, annually costing an estimated $3 billion on prevention and treatment (Griffin, 1997). BRD poses an even greater threat in the beef-on-dairy sector. For example, a study of 800 cattle in a Kansas feedlot found that, of cattle treated for BRD, beef-on-dairy crossbred cattle were twice as likely to have higher case fatality compared to beef-breed cattle (Theurer et al., 2020). This makes the integration of beef-on-dairy calves into feedlots a major challenge.

Calves born on commercial dairies face greater copper (**Cu**) exposure than those born to beef cows due to several factors. Dairy cows are commonly fed over 17 mg Cu/kg diet DM (Castillo et al., 2013), which is 6 mg greater than the NASEM (2021) recommendations for moderate-producing dairy cows and 8 mg greater than for high-producing cows. Subsequently, cases of Cu toxicosis in dairy cows have increased in recent years (Kendall et al., 2015). Additionally, because Cu is transported to the fetus via the placenta (McArdle and Erlich, 1991), calves born to dairy cows are at risk of high liver Cu concentrations at birth. Fortification of the milk replacer and starter with Cu likely explain why Puschner et al. (2004) found dairy calves had liver Cu concentrations 125 to 200 mg Cu/kg liver DM greater than beef-breed calves.

While maintaining adequate Cu status is essential for supporting growth, immune function, and antioxidant capacity, excess Cu can cause oxidative stress. As the ability to safely store Cu within the cell is exceeded, free Cu^+^ ions are released, which participate in the Haber–Weiss reaction to generate reactive oxygen species (Bremner, 1998). This contributes to inflammation and may impair immune function. In mice fed high-Cu diets, the cellular immunity, humoral immunity, and proinflammatory cytokine production in response to stimuli were impaired (Pocino et al., 1991).

Given the health challenges beef-on-dairy calves face, their increased Cu exposure, and the few previous studies conducted in mice, the objective of this study was to determine the impact of excess liver Cu concentration in beef-on-dairy calves on their response to a BRD challenge. We hypothesized that steers with excess liver Cu will have impaired ability to overcome disease and exhibit greater disease severity.

## Materials and Methods

All experimental procedures were approved by the Iowa State University Institutional Animal Care and Use Committee (log no. 22-213) and followed standards described by the Guide for the Care and Use of Animals in Research and Teaching, fourth edition (FASS, 2020).

### Animals and experimental design

Twenty-six beef-on-dairy crossbred steers (95.2 ± 7.2 kg; ~8 wk old) were delivered to the Iowa State University Beef Nutrition Farm in Ames, IA on May 18th, 2023 (day −120). Steers were purchased from a single calf-raiser who had sourced the calves from multiple dairies. Steers were blocked by body weight (**BW**) into pens of six to eight head, and one pen from each weight block was assigned to either adequate- (**ADE**) or HIGH-Cu diets to create two groups of steers with distinct liver Cu groupings. Upon arrival, steers were dewormed with Safe-Guard oral suspension (Merck Animal Health, Madison, NJ). A pelleted diet (**Table 1**) containing 10 or 20 mg Cu/kg diet DM for ADE and HIGH, respectively, was fed until steers were transitioned to a total mixed ration (**TMR**; **Table 2**) plus ad libitum hay on day −73. This diet contained 0 or 10 mg Cu from Cu sulfate/kg diet DM for ADE and HIGH, respectively. All steers were treated with Draxxin (Zoetis, Parsippany, NJ) on day −95 due to signs of respiratory outbreak. All steers received a zeranol implant (Ralgro, Merck Animal Health) on day −81. Liver biopsies were collected from all animals on day −39 to confirm Cu status aligned with treatment assignments, and *n* = 13 steers were used for each treatment. Liver Cu averaged 290 ± 96 and 603 ± 90 mg Cu/kg liver DM for ADE and HIGH, respectively.

**Table 1.** Analyzed composition of pelleted diet fed from day −120 to day −73

Analyzed composition^1^	High-Cu pellet	Low-Cu pellet
Crude protein, % DM^2^	25.3	25.1
Neutral detergent fiber, % DM^2^	16.9	16.5
Ether extract, % DM^2^	3.8	4.0
Sulfur, % DM^3^	0.45	0.42
Molybdenum, mg/kg DM^3^	1.08	1.59
Copper, mg/kg DM^4^	22.8	8.8
Zinc, mg/kg DM^4^	54.0	56.5
Iron, mg/kg DM^4^	184	183

^1^Weekly samples were collected, dried in a forced air oven at 70 °C, ground, and composited by month for analysis.

^2^Analysis performed by Dairyland Laboratories (Arcadia, WI).

^3^Analysis performed via inductively coupled plasma–mass spectrometry by the Iowa State University Veterinary Diagnostic Laboratory (Ames, IA).

^4^Analysis performed via ICP–OES (ICP Optima 7000 DV, Perkin Elmer, Waltham, MA).

**Table 2.** Diet composition of total mixed ration fed from day −73 to day 14

Ingredient	% of diet DM
Hay	15
Corn	15
Corn silage	15
Dried distillers grains	18.06
Sweet bran^1^	35
Cu premix^2^	5
Trace mineral premix^3^	0.0204
Limestone	1.5
Salt	0.31
Vitamin A & E premix^4^	0.1
Rumensin 90	0.0135
Analyzed composition	
Crude protein, %^5^	18.4
Neutral detergent fiber, %^5^	33.1
Ether extract, %^5^	5.2
Sulfur, %^6^	0.31
Molybdenum, mg/kg DM^6^	0.97
Copper, mg/kg DM^7^	4.8
Zinc, mg/kg DM^7^	67.5
Iron, mg/kg DM^7^	211

^1^Branded wet corn gluten feed (Cargill Milling, Blair, NE).

^2^Cu treatments were included as a dried distillers grains-based premix that replaced dried distillers grains in the diet. Treatments included: no supplemental Cu (ADE), 10 mg Cu/kg diet DM (HIGH), and 5 mg Cu/kg diet DM (fed to both treatments during disease challenge).

^3^Trace mineral premix was formulated to supplement all trace minerals other than Cu at NASEM (2016) recommendations.

^4^Vitamin A & E premix provided 2,200 IU vitamin A and 25 IU vitamin E/kg diet DM.

^5^Analysis of ADE TMR composite by Dairyland Laboratories (Arcadia, WI).

^6^Analysis of ADE TMR composite by the Iowa State University Veterinary Diagnostic Laboratory (Ames, IA).

^7^Analyzed Fe, Zn, and Cu represent ADE dietary treatment with no supplemental Cu and were analyzed via ICP–OES (ICP Optima 7000 DV, Perkin Elmer, Waltham, MA). All treatments were supplemented Zn and Fe to meet NASEM (2016) recommendations. Copper treatments were supplemented in addition to Cu in the basal diet.

Steers were transported for 6 h on day −1 to mimic arrival stress and delivered to the Animal Resource Station in Ames, IA. Starting on day 0, all steers were supplemented with 5 mg Cu from Cu sulfate/kg diet DM, regardless of treatment. Diet composition remained unchanged. Treatments were housed in two separate, but adjacent, pens. Steers were infected with 10^4^TCID_50_ bovine respiratory syncytial virus (**BRSV**; strain 375) on day 0 via aerosol inoculation using methods previously described by Sacco et al. (2012). On day 5, steers were intratracheally infected with 5 × 10^8^ colony-forming unit (**CFU**) *Mannheimia haemolytica*, prepared and administered as previously described (Capik et al., 2015; Hong et al., 2024).

### Sample collection and analytical procedures

Weekly pellet, TMR, and ingredient samples were collected and dried in a forced air oven at 70 °C for 48 h to determine DM content, after which they were ground through a 2 mm screen (Retsch Zm100 grinder; Retsch GmBH, Haan, Germany). Samples were then composited by month for analysis of crude protein (AOAC, 1995b; method 990.3), neutral detergent fiber (AOAC, 2005; method 2002.04), and ether extract (AOAC, 1995a; method 930.29) by Dairyland Laboratories (Arcadia, WI). Sulfur and molybdenum concentrations were measured via inductively coupled plasma–mass spectrometry by the Iowa State University Veterinary Diagnostic Laboratory (Ames, IA).

Liver biopsies were collected 2 h post-feeding on day −39 and day 13 following methods described by Engle and Spears (2000). Samples were transported to the laboratory on ice and stored at −20 °C until drying in a forced air oven at 70 °C until completely dry, approximately 7 d.

Blood was collected prior to feeding, at approximately 0700 h, on days 0, 5, 7, 10, and 13 via jugular venipuncture in serum separator tubes and plasma K_2_EDTA tubes (Becton Dickinson, Franklin Lakes, NJ). Samples were transported into the lab and centrifuged at 1,000 ×* g* for 20 min at 4 °C. Plasma and serum were harvested and stored at −20 and −80 °C, respectively, until further analysis. Serum haptoglobin concentration on days 0, 7, and 13 was quantified using a commercial, bovine-specific, enzyme-linked immunosorbent assay (ELISA) kit (Catalog number: E-10HPT; Immunology Consultants Laboratory, Inc., Portland, OR). Total antioxidant capacity in serum samples from days 0, 7, and 14 was determined using a commercial ferric reducing antioxidant power (**FRAP**) assay (Catalog number: K043-H1; Arbor Assays, Ann Arbor, MI), in which a ferric complex was added to the sample, and the reduction of this complex to a ferrous complex by antioxidants present in the sample was measured through a colorimetric reaction. A large animal routine chemistry profile was run on serum samples from day 0 by the Iowa State University Veterinary Diagnostic Laboratory (Ames, IA). Analytes included aspartate aminotransferase, gamma-glutamyl transferase, alkaline phosphatase (**ALP**), creatinine, creatine kinase, total bilirubin, albumin, total protein, glucose, and blood urea nitrogen.

After drying, feed and liver samples were acid digested (CMES MARSXpress, Matthews, NC) with trace mineral grade nitric acid as described by Richter et al. (2012). Plasma, liver, and TMR composites were prepared and analyzed for Cu concentration via inductively coupled plasma–optical emission spectrometry (**ICP–OES**; ICP Optima 7000 DV, Perkin Elmer, Waltham, MA) as described by Pogge and Hansen (2013).

To determine gut permeability, 180 mM chromium–EDTA (**Cr–EDTA**) was prepared as previously described (Wood et al., 2015), and 500 mL were administered orally to each steer prior to infection on day 0 as well as on day 7. Four hours after dosing with Cr–EDTA, at approximately 1400 h, blood samples were collected into K_2_EDTA plasma tubes via jugular venipuncture. Plasma was harvested as described previously, and Cr concentrations were measured via ICP–OES as described previously with slight modifications: plasma was diluted 1:3 in 5% trace mineral grade nitric acid, and a stock solution (TruQ Chromium; PerkinElmer) with a concentration of 1,000 µg Cr/mL was used to create standards to compare unknown samples against.

Clinical signs of disease were evaluated daily from day 0 (preinfection) to day 14 by a single trained observer according to the DART system (depression, appetite, respiratory changes, and temperature; Griffin et al., 2010). In addition, rectal temperature was measured on days 0, 5, 7, 10, and 13.

Thoracic ultrasonography (**TUS**) was performed to determine lung consolidation in response to infection on days 0, 5, 7, 10, and 13 using an IBEX EVO (E.I. Medical Imaging, Loveland, CO) with the L7HD linear transducer probe (5 to 9 MHz) set to a depth of 11.6 cm. Ultrasound procedures were conducted as previously described (Hong et al., 2024), with five locations on the left (intercostal space 19, 18, 14, 10, and 9) and right (intercostal space 23, 22, 18, 19, and 14) sides used for scoring. A scoring system was modified from Rademacher et al. (2013), where fewer than five pleural defects are a score of 0, and five or more pleural defects but no consolidation receives a score of 1. Lung consolidation is scored 2 to 4 depending on the maximum depth of consolidation: a score of 1 is less than or equal to 2 cm, a score of 2 is between less than or equal to 2 cm but less than 4 cm, and a score of 3 is greater than or equal to 4 cm of consolidation. A trained individual was responsible for reading and scoring ultrasound images.

Bronchoalveolar lavage (**BAL**) fluid was collected on days 0 and 7 as previously described (Guerra-Maupome et al., 2019). Briefly, a silicone large-animal BAL catheter (10 mm × 2.5 mm × 240 cm long, MILA International, Inc., Florence, KY) was inserted into the nose and passed through the trachea until reaching the bronchus. A syringe was used to deliver 60 mL of sterile saline to the lower respiratory tract, followed by 30 mL of air and immediate suction to obtain wash from the lower airway. This procedure was performed twice more, and BAL fluids were pooled within animal and transported to the laboratory on ice before filtration through sterile gauze. Filtered fluid was centrifuged at 2,000 × *g* for 10 min, after which samples were stored in 24% glycerol for bacterial preservation. To determine bacterial recovery, preserved BAL samples were thawed and centrifuged at 3,000 × *g* for 10 min. Pelleted bacteria were then resuspended in 100 µL of sterile saline, plated on blood agar plates, and cultured for 2 d prior to counting *M. haemolytica* bacterial colonies. BAL cell pellets from each sample were also stored in TRIzol reagent (Invitrogen, Carlsbad, CA), and total RNA was isolated via RNeasy spin columns according to the manufacturer instructions (QIAGEN, Hilden, Germany), and 500 ng of RNA was used to synthesize cDNA. Power SYBR Green Master Mix (Applied Biosystems, Carlsbad, CA) was used to perform qPCR as previously described, utilizing a ThermoFisher (Waltham, MA) Quant Studio 5 Real-Time PCR machine. Primer information for target genes relating to Cu metabolism is displayed in **Table 3**; *RPS9* was utilized as a reference gene. Target genes included copper transporter 1, lysyl oxidase, matrix metallopeptidase 9, metallothionein 2a, and nuclear factor erythroid-2–related factor 2. Primers for qPCR were validated for specificity and efficiency by in silico analysis and melt curve assessment.

**Table 3.** Forward and reverse primers for quantitative real-time polymerase chain reaction in cells recovered from BAL samples

Gene	Function	Primer sequence (5′–3′)	Accession no.
*CTR1* ^1^	Primary route of copper import	F: AAGAGTCCTGGAGGTGTG	NM001100381.1
		R: GGTCAGATGAAGTGGTTGG	
*LOX* ^2^	Copper-dependent; extracellular matrix development and repair	F: AGCTATTTGGTGCCTGAGTC	NM173932
		R: ATATGCGTGATGTCCTGTGTAG	
*MMP9* ^3^	Zinc dependent; collagen degradation	F: GGTATGGGTAATGAAATTGATGTG	XM592003.4
		R: TCAGCAGCGATGTAGTAGTG	
*MT2A* ^4^	Copper storage and protection from oxidative stress	F: GACCCCAGCCTCCAGTTCAGCTC	NM001075140.1
		R: CTTTGCATTTGCAGGAGCCGGC	
*NRF2* ^5^	Antioxidant	F: CCCAGTCTTCACTGCTCCTC	NM001011678
		R: TCAGCCAGCTTGTCATTTTG	
*RPS9* ^6^	Housekeeping gene	F: CCTCGACCAAGAGCTGAAG	DT860044
		R: CCTCCAGACCTCACGTTTGTTC	

^1^Copper transporter 1.

^2^Lysyl oxidase.

^3^Matrix metallopeptidase-9.

^4^Metallothionein 2A.

^5^Nuclear factor erythroid-2–related factor 2.

^6^Ribosomal protein subunit 9.

Nasopharyngeal swabs were collected on days 0, 5, 7, 10, and 13 to determine *M. haemolytica* bacterial load. The swabs were stored in sterile 5 mL microtubes and transported to the laboratory on ice. In brief, 2 mL 1× minimum essential medium (Gibco) was added to each tube and vortexed. Approximately 1.2 mL of the washed media was recovered and transferred to a sterile microcentrifuge tube and was used as the inoculum to obtain counts following a previously published protocol with slight modifications (Slate et al., 2021). Briefly, 100 µL of the inoculum was diluted 1:100 and 1:1,000 to ensure countable colonies. One hundred microliters of each dilution were spread onto blood agar plates (Remel, Lenexa, KS) and incubated at 37 °C for 24 h, following the standard incubation protocol for bacterial culturing (Briggs et al., 2012; Slate et al., 2021). Colonies with typical *M. haemolytica* morphology were counted from one of the dilutions, and the CFU/mL was calculated.

### Statistical analysis

The MIXED procedure of SAS 9.4 was used to analyze haptoglobin, FRAP, plasma trace mineral, nasopharyngeal swab bacterial load, and rectal temperature data with the fixed effect of Cu treatment and day postinfection as repeated measure. Bronchoalveolar lavage bacterial recovery, gene expression, markers of liver damage, and plasma Cr concentration were also analyzed in the MIXED procedure of SAS with the fixed effect of Cu treatment within day (day 0 or day 7). Because not all samples analyzed for Cr were collected precisely 4 h after dosing, the exact time between dose and sample collection was used as a covariate in the analysis of plasma Cr concentrations. Haptoglobin, nasopharyngeal bacterial load, and BAL bacterial load were transformed using the natural logarithm to meet assumptions for normality; LSMEANS and SEM are back-transformed for discussion. Clinical scores were analyzed in the GLIMMIX procedure of SAS as categorical values with the fixed effect of Cu treatment and day postinfection as repeated measure. At various timepoints throughout the disease challenge, three steers (one ADE and two HIGH) required treatment and were removed from the study after being treated; data collected from these animals prior to treatment were included in the statistical model. Cook’s D statistic was used to test for outliers with a cutoff value of 0.5; however, none were detected.

## Results

### Clinical outcomes

Liver Cu averaged 290 ± 96 and 603 ± 90 mg Cu/kg liver DM for ADE and HIGH, respectively. Over the course of the disease challenge, three steers were removed from the study and treated due to health concerns. Reasons for removal included high rectal temperature, difficulty breathing, and lethargy. One HIGH steer was removed on day 3 postinfection, treated, and euthanized 6 days postinfection. Another HIGH steer was removed and treated on day 5 and was not inoculated with *M. haemolytica.* One ADE steer was removed after sampling on day 7. Any steer that was removed from the study was included in statistical analysis up until the point of removal.

### Bacterial recovery

*Mannheimia haemolytica* was only recovered from one animal on day 0 (HIGH; **Table 4**); therefore, statistical analysis of bacterial recovery from nasopharyngeal swabs was conducted only on days 5, 7, 10, and 13. Bacterial recovery from nasopharyngeal swabs was not affected by treatment (*P = *0.95) or treatment × day (*P* = 0.23) but was affected by day (*P* < 0.01), where recovery was highest on day 5 (*P* < 0.01), with all other days postinfection not differing (*P *≥ 0.14). *Mannheimia haemolytica* recovery from BAL samples on day 0 and day 7 was not affected by treatment (*P *≥ 0.14).

**Table 4.** Effect of excess liver Cu and day postinfection on *M. haemolytica* recovery from BAL

Item	Treatment^1^		SEM^2^	Treatment *P*-value	Day	SEM	Day *P*-value
	ADE	HIGH					
Nasopharyngeal swab *M. haemolytica* count, CFU/mL^3^				0.95			<0.01
			
Day 0	0	0	–		0	–	
Day 5	19	2	35		7^b^	8	
Day 7	929	3,695	5,976		1,853^a^	2,042	
Day 10	22,402	6,042	34,709		11,634^a^	13,066	
Day 13	898	10,306	16,776		3,041^a^	3,426	
BAL *M. haemolytica* count, CFU/mL^4^							
			
Day 0	119	12	127	0.14	37	29	–
Day 7	3	8	14	0.69	5	6	

^1^Treatments included HIGH liver Cu and ADE liver Cu (603 and 279 mg Cu/kg liver DM prior to challenge, respectively).

^2^Highest SEM of either treatment.

^3^Nasopharyngeal swabs were collected on days 0, 5, 7, 10, and 13. Bacterial recovery was analyzed with the main effects of treatment, day, and their interaction. *P*-values for main effects of treatment and day are shown; treatment × day was not significant (*P* = 0.23). For the day effect, values with unlike superscripts differ by day (*P* ≤ 0.05).

^4^BAL samples were collected on days 0 and 7, and data were analyzed with the main effect of treatment within day.

### Signs of clinical disease

**Figure 1.**
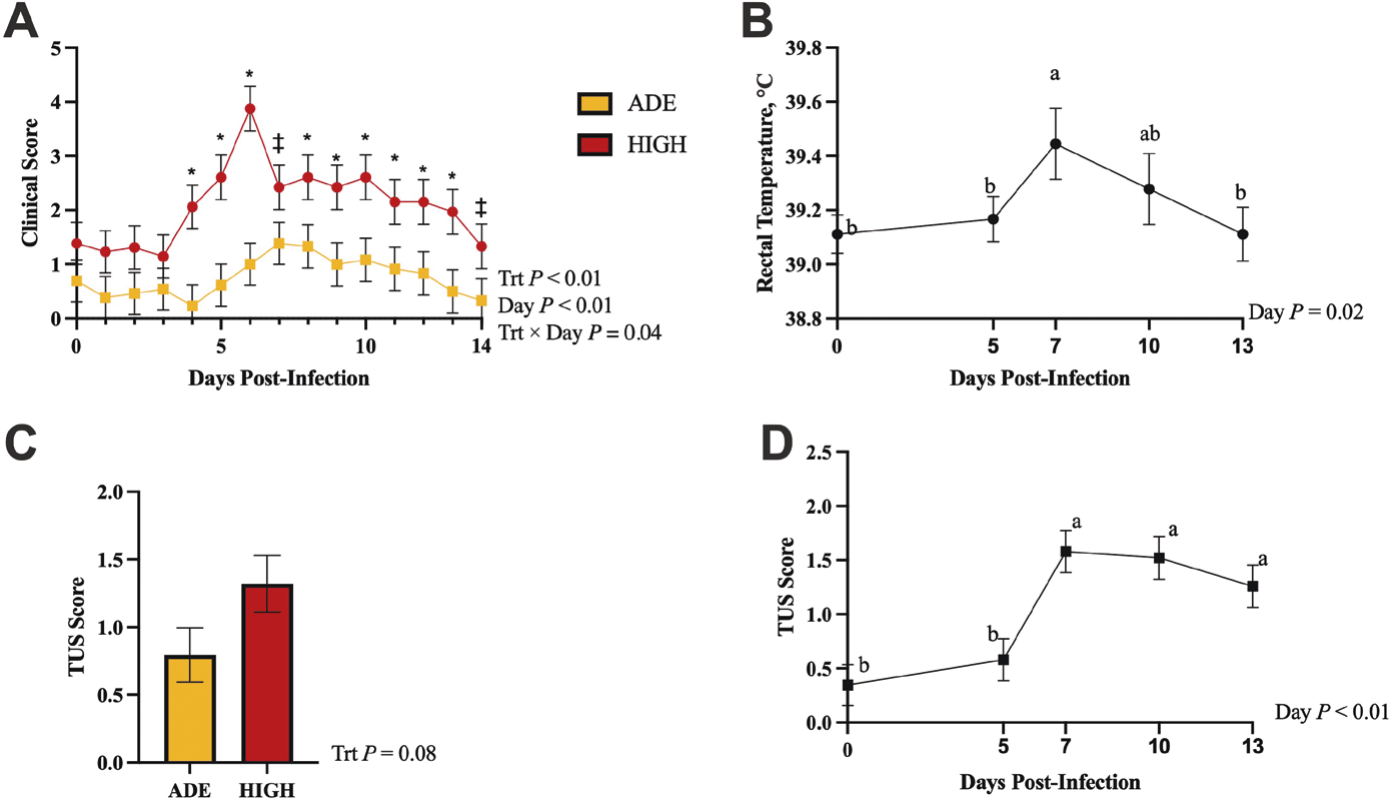
Effect of excess liver Cu on clinical disease scores (A), rectal temperatures (B), and TUS scores (C and D) over the course of disease challenge in beef-on-dairy crossbred steers. Steers were infected with BRSV on day 0 and *Mannheimia haemolytica* on day 5. (A) Within-day postinfection; * indicates significant difference between ADE and HIGH (*P* ≤ 0.05) and ‡ indicates tendencies (0.05 < *P* ≤ 0.10). (B) Data points with unlike superscripts differ by day (*P* ≤ 0.05). (C) Scores tended to be affected by treatment (*P* = 0.08). (D) Data points with unlike superscripts differ by day (*P* ≤ 0.05) and were affected by day.

Clinical scores are presented in **Figure 1** and were affected by treatment × day (*P* = 0.04), where both treatments had similar scores from days 0 to 3 (*P* ≤ 0.13), but HIGH steers had greater clinical scores starting on day 4, which peaked on day 6 (*P* < 0.01) and remained greater than those of ADE steers through day 14 (*P* ≤ 0.08). Clinical scores in ADE steers increased gradually from days 4 to 7, with day 7 being greater than day 4 (*P* > 0.01) and days 5 and 6 being intermediate. From days 8 to 14, ADE steers gradually return to preinfection scores, with day 8 being greater than day 14 (*P *= 0.02) and days 9 to 13 being intermediate. Clinical scores were also affected by treatment (*P *< 0.01), with HIGH having greater clinical scores than ADE.

Rectal temperatures are presented in **Figure 1** and were not affected by treatment or treatment × day (*P* ≤ 0.27). Rectal temperatures were affected by day (*P* = 0.02), where they are highest on day 7 (*P* ≤ 0.04) of the disease challenge and return to preinfection temperatures on day 13, with day 10 being intermediate.

Lung consolidation scores obtained from TUS are displayed in **Figure 1** and were affected by day (*P* < 0.01), where scores on days 0 and 5 were similar (*P* = 0.25) and lower than those on days 7, 10, and 13 postinfection (*P* < 0.01). HIGH steers tended to have greater TUS scores than ADE overall (*P *= 0.08).

### Trace mineral concentrations

By design, liver Cu concentrations differed by treatment on both timepoints, where HIGH had greater liver Cu concentrations then ADE (*P* < 0.01; **Table 5**).

**Table 5.** Liver Cu concentrations of ADE and HIGH treatments before and after disease challenge

Item	Treatment^1^		SEM^2^	Treatment *P*-value
	ADE	HIGH		
Liver Cu, mg/kg DM^3^				
Day −39^4^	279	603	27.8	<0.01
Day 13^5^	250	516	32.6	<0.01

^1^Treatments included HIGH liver Cu and ADE liver Cu (603 and 279 mg Cu/kg liver DM prior to challenge, respectively).

^2^Highest SEM of either treatment.

^3^Liver Cu measured via ICP–OES (ICP Optima 7000 DV; Perkin Elmer, Waltham, MA).

^4^The ranges for ADE and HIGH were 132–420 and 488–809, respectively.

^5^The ranges for ADE and HIGH were 158–457 and 361–838, respectively.

**Figure 2.**
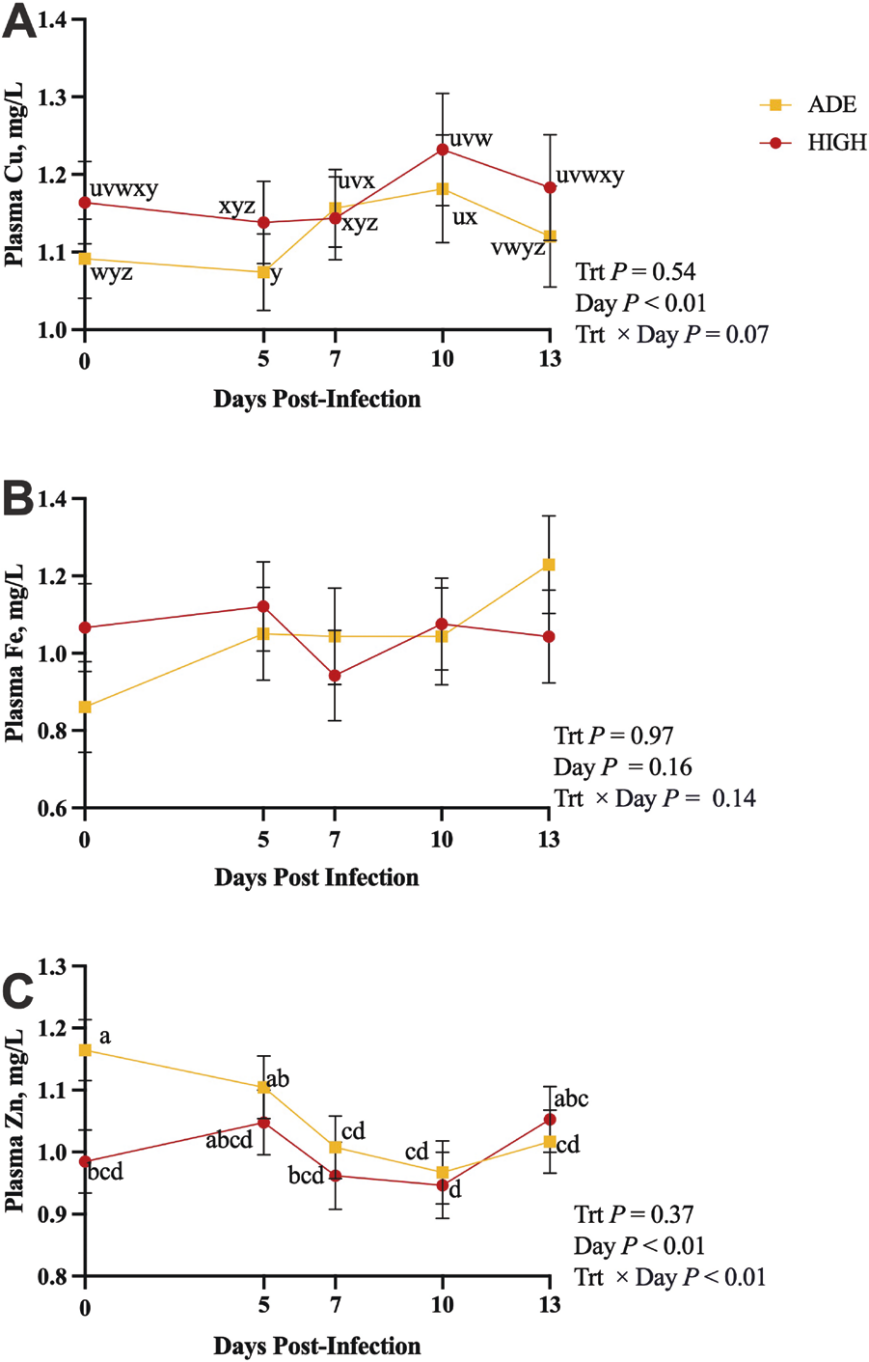
The effect of excess liver Cu, day relative to infection, and their interaction on plasma Cu (A), plasma Fe (B), and plasma Zn (C) in blood samples collected on days 0, 5, 7, and 10 postinfection. Steers were infected with BRSV on day 0 and *Mannheimia haemolytica* on day 5. (A) Terms with unlike superscripts tend to differ (*P* ≤ 0.1). (C) Terms with unlike superscripts differ (*P* ≤ 0.05).

Plasma Cu tended to have a treatment × day interaction (*P* = 0.07; **Figure 2**), where both treatments were similar (*P* ≥ 0.34) and remained consistent from day 0 to day 5 (*P* ≥ 0.25). Plasma Cu in ADE steers increased from day 5 to day 7 (*P* < 0.01), remained consistent on day 10 (*P *= 0.43), and decreased on day 13 (*P* = 0.04). HIGH increased from day 5 to day 10 (*P* = 0.03), with day 7 being intermediate, and remained steady on day 13 (*P* 0.31). Plasma Zn was affected by treatment × day (*P* < 0.01), where ADE had greater plasma Zn than HIGH on day 0 (*P* = 0.01). ADE remained steady through day 5 (*P* = 0.18) and decreased on days 7, 10, and 13 (*P* ≤ 0.03). HIGH did not change from day 0 to day 10 (*P* ≥ 0.18) and had increased plasma Zn from day 10 to day 13 (*P* = 0.03). Plasma Fe was not affected by treatment, day, or treatment × day (*P* ≥ 0.14).

### Markers of antioxidant status

**Figure 3.**
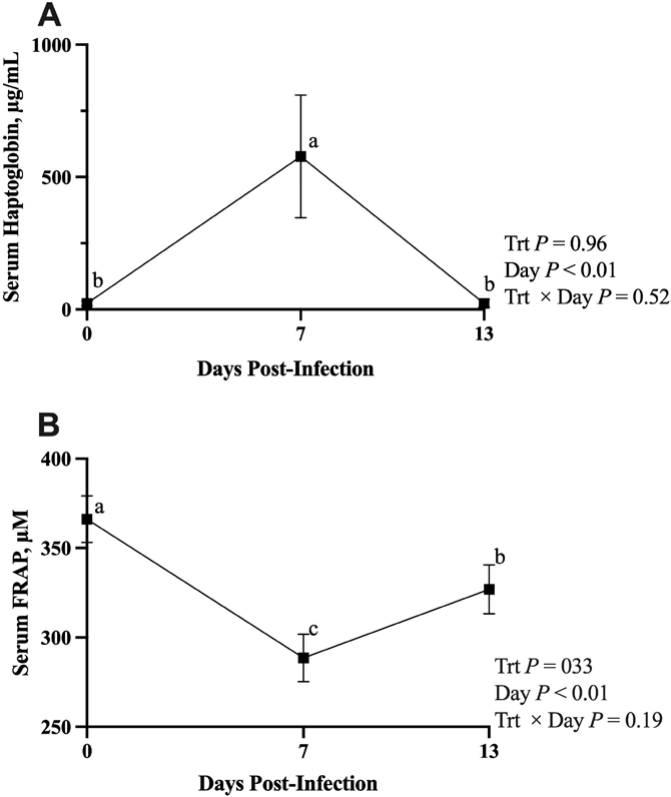
The effect of day postinfection on (A) serum haptoglobin concentration and (B) serum FRAP in beef-on-dairy steers. Steers were infected with BRSV on day 0 and *Mannheimia haemolytica* on day 5. Within a panel, data points with unlike superscripts differ by day (*P* ≤ 0.05).

FRAP was not affected by treatment or treatment × day (*P* ≥ 0.19; **Figure 3**). Day was significant (*P* < 0.01), where days 0, 7, and 13 were all different from each other (*P* ≥ 0.02), with day 0 being the lowest, day 7 the greatest, and day 13 intermediate. Haptoglobin was not affected by treatment or treatment × day (*P* ≥ 0.52) but was affected by day (*P* < 0.01), where day 0 and 13 were not different (*P* = 0.99) with the peak on day 7 (*P* ≤ 0.01).

### Serum metabolite profile

Excess liver Cu prior to disease challenge had no effect on aspartate aminotransferase, gamma-glutamyl transferase, creatinine, creatine kinase, total protein, or blood urea nitrogen (*P* ≥ 0.42; **Table 6**). Steers with adequate liver Cu had greater concentrations of total bilirubin, albumin, and glucose (*P* ≤ 0.03) and tended to have greater concentrations of ALP (*P* = 0.07) than HIGH steers.

**Table 6.** Effect of excess liver Cu on serum metabolite profile

Item^2^	Treatment^1^		SEM^3^	Treatment *P*-value
	ADE	HIGH		
Aspartate aminotransferase, IU/L^4^	4.45	4.41	5.790	0.65
Gamma-glutamyl transferase, IU/L	35.1	35.8	1.48	0.74
Alkaline phosphatase, IU/L	183.9	148.7	13.26	0.07
Creatinine, mg/dL	0.84	0.88	0.026	0.42
Creatine kinase, IU/L^4^	160.0	143.0	18.77	0.50
Total bilirubin, mg/dL	0.55	0.45	0.022	<0.01
Albumin, g/dL	3.00	2.85	0.045	0.03
Total protein, g/dL	7.33	7.39	0.165	0.79
Glucose, mg/dL	68.7	49.1	3.32	<0.01
Blood urea nitrogen, mg/dL	8.69	8.15	0.402	0.35

^1^Treatments included HIGH liver Cu and ADE liver Cu (603 and 279 mg Cu/kg liver DM prior to challenge, respectively).

^2^Serum samples collected immediately prior to infection were sent to the Iowa State University Clinical Pathology Laboratory (Ames, IA), where markers of liver damage were measured.

^3^Highest SEM of either treatment.

^4^Estimates were transformed using the natural logarithm to meet assumptions for normality. Presented data are back-transformed LSMEANS and SEM.

### Gene expression from BAL cells

On day 0, there were no differences in expression between treatment for any gene (*P* ≥ 0.36; **Table 7**). On day 7, HIGH steers tended to have greater *NRF2* (an oxidative stress gene) expression than ADE steers (*P* = 0.06). Expression of all other genes was similar between treatments (*P* ≥ 0.18).

**Table 7.** Effect of excess liver Cu on relative expression^1^ of Cu metabolism and anti-inflammatory genes in cells recovered from BAL on days 0 and 7 of disease challenge

Item	Treatment^2^		SEM^3^	Treatment *P*-value
	ADE	HIGH		
Day 0				
* CTR1* ^4^	1.153	0.981	0.1819	0.65
* LOX* ^5^	1.154	1.453	0.3420	0.90
* MMP9* ^6^	2.149	6.510	3.5257	0.36
* MT2A* ^7^	1.056	1.262	0.8915	0.62
* NRF2* ^8^	1.097	0.968	0.0803	0.66
Day 7				
* CTR1* ^4^	1.101	1.311	0.1368	0.18
* LOX* ^5^	1.421	1.296	0.3670	0.83
* MMP9* ^6^	2.479	5.413	2.7624	0.35
* MT2A* ^7^	1.283	0.910	0.2404	0.58
* NRF2* ^8^	1.109	1.328	0.0751	0.06

^1^Relative expression was determined using the 2^-∆∆CT method, where CT values were normalized to the housekeeper gene (*RPS9*). Then, the average ∆CT value from the ADE treatment was subtracted from the ∆CT value of each target to obtain ∆∆CT. Statistical analysis was performed on ∆CT values.

^2^Treatments included HIGH liver Cu and ADE liver Cu (603 and 279 mg Cu/kg liver DM prior to challenge, respectively).

^3^Highest SEM of either treatment.

^4^Copper transporter 1.

^5^Lysyl oxidase.

^6^Matrix metallopeptidase-9.

^7^Metallothionein 2A.

^8^Nuclear factor erythroid-2–related factor 2.

### Gastrointestinal tract permeability

**Figure 4.**
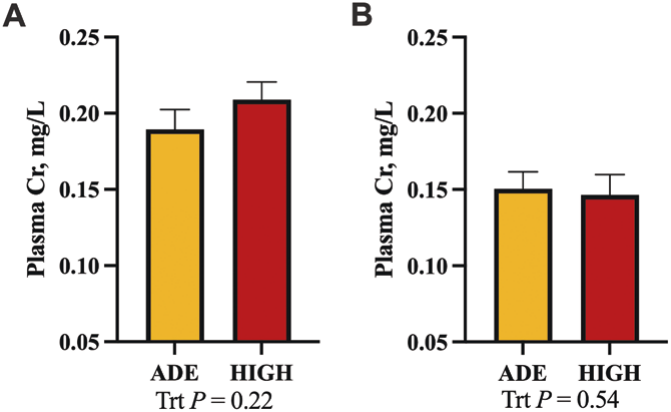
The effect of excess liver Cu on gut permeability in beef-on-dairy crossbred steers on day 0 (A) and day 7 (B) postinfection. Steers were infected with BRSV on day 0 and *Mannheimia haemolytica* on day 5. Chromium EDTA was used as a marker for gut permeability.

Chromium concentrations did not differ between treatment at either timepoint (*P *≥ 0.22; **Figure 4**).

## Discussion

Prior to challenge, liver Cu concentrations averaged 279 (range 129 to 420) and 603 (range 488 to 809) mg Cu/kg liver DM for ADE and HIGH, respectively. Based on the “adequate” liver Cu range of 125 to 600 mg Cu/kg liver DM proposed by Kincaid (1999), ADE steers were in the middle of the adequate range and HIGH steers were at the upper threshold of adequate. HIGH steers showed more signs of clinical disease from day 4 postinfection through the end of the trial, as evidenced by their greater clinical scores. HIGH also had the greatest clinical scores on day 6 postinfection, which aligns with the expected peak in severity of disease symptoms (Porter et al., 2021). Calves in ADE experienced much less severe increases in clinical scores and returned to preinfection scores by the end of the 13-d study, while HIGH had not. Further, HIGH tended to have greater TUS scores, indicating increased damage and consolidation in the lungs in response to disease.

Plasma Cu and Zn responded differently to disease between treatments, where HIGH steers did not change over the course of disease, and ADE steers exhibited fluctuations in plasma Cu and Zn that are characteristic of the classical nutritional immunity response that has been well documented by other studies. In cattle, both Galarza et al. (2021) and Hong et al. (2024) noted decreases in plasma Zn concentrations following *M*. *haemolytica* infection, and Orr et al. (1990) found decreased plasma Zn following bovine rhinotracheitis infection. This decrease in plasma Zn in response to infection and inflammation is thought to starve pathogens of Zn, slowing the progression of disease. More recently, it has been suggested that the decrease in plasma Zn concentrations during infection is part of a cell signaling process in which Zn is recruited to tissues to serve as a signal to enhance immune cell activation (Kim and Lee, 2021). Meanwhile, plasma Cu increases with the nutritional immunity response, as Cu-containing ceruloplasmin is released as part of the acute phase response (Orr et al., 1990). Given the lack of nutritional immunity response in the HIGH treatment and their greater clinical scores, excess liver Cu appears to hinder the response to infection, resulting in more severe signs of clinical disease.

Despite their increased clinical scores and lack of nutritional immunity response, the HIGH treatment did not have greater haptoglobin concentrations than ADE. In general, serum haptoglobin concentrations were widely variable across both treatments. Disease challenge resulted in haptoglobin concentrations on day 7 that were over 120-fold greater than those on day 0, suggesting that the disease challenge caused inflammation. Hong et al. (2024) infected beef steers weighing approximately 315 kg with *M. haemolytica* and noted haptoglobin concentrations peaked at approximately 18 µg/mL 2 d postinfection. Though not a perfect comparison because the steers in the present study experienced a BRSV and *M. haemolytica* coinfection model and were 90 kg lighter, the steers in the present study peaked at over 500 µg/mL 2 d following *M*. *haemolytica* infection. Even though haptoglobin was not affected by liver Cu, understanding basal inflammatory rates of beef-on-dairy animals compared to beef-breed animals may be important to best manage them in the feedlot.

FRAP was measured as an indicator of total circulating antioxidant capacity. Both treatments have decreased antioxidant capacity on day 7, which is concurrent with the increased inflammation at the same timepoint. Contrary to our hypothesis, excess liver Cu did not lead to decreased FRAP; however, while not statistically significant, it is interesting to note that the decrease in FRAP from day 0 to day 7 is more pronounced in HIGH than in ADE (28% decline in HIGH vs. 14% in ADE). This may suggest excess Cu influences antioxidant status, but more research is needed to confirm this. In our prior transit stress work (Deters and Hansen, 2022), FRAP declines and markers of inflammation increase in response to trucking, supporting a relationship between FRAP and inflammation. If excess Cu does decrease FRAP, it may follow that excess Cu increases inflammation. Research on antioxidant capacity and inflammation in similar animals and challenge models to the present study is lacking, and more research is needed to compare the inflammatory response between beef-on-dairy and beef-breed calves.

Steers with excess liver Cu have a different blood metabolite profile than those with normal Cu concentrations. HIGH steers had lesser serum albumin concentrations than ADE steers. Both treatments were within previously reported reference ranges for healthy adult cattle (Knowles et al., 2000). Wilson’s disease decreases serum albumin as a function of liver damage induced by excess liver Cu (Boga et al., 2017). While HIGH Cu cattle in the present study are still within the normal range, it is possible they had decreased albumin due to liver dysfunction caused by excess Cu. Both treatments had total bilirubin concentrations well in excess of previously reported average concentrations (Doornenbal et al., 1988; Mohri et al., 2007). Bilirubin is formed from the breakdown of hemoglobin in red blood cells and excreted in the bile (Wang et al., 2006), and elevation can be an indicator of liver damage, bile duct obstruction, or greater red blood cell turnover. ADE steers had greater total bilirubin concentrations than HIGH, but the mechanisms driving this response are unknown.

ALP concentrations for both treatments are on the upper threshold of the healthy reference range (Aiello and Moses, 2016); however, Knowles et al. (2000) have shown that serum ALP concentrations are highest at birth and decrease steadily with age. The tendency for ADE calves to have increased ALP may be a function of Zn status, as ALP is a Zn-dependent enzyme (Yamaguchi and Yamaguchi, 1986). The fact that the ADE treatment had greater circulating Zn than HIGH may have also meant ADE had more Zn available to support ALP in circulation.

Intriguingly, HIGH steers had markedly lower concentrations of circulating glucose, which could be attributed to the increased glucose requirements of an activated immune system. Kvidera et al. (2017) administered lipopolysaccharide (**LPS**), a potent proinflammatory agent, to lactating Holstein cows and determined acute inflammation caused hypoglycemia. The authors further quantified the glucose requirement of LPS-challenged cattle and found these animals required over 1 kg of glucose to be infused over 720 min to maintain serum glucose concentrations equivalent to non-LPS-administered controls (Kvidera et al., 2017). If the HIGH steers had greater disease severity or inflammation than ADE, the immune system may be utilizing glucose at a greater rate than it can be synthesized in the liver, leading to decreased glucose in circulation compared to ADE. Also, circulating glucose in ruminants is largely driven by hepatic gluconeogenesis (Bergman, 1976), so differences in serum glucose may be a result of differences in liver function. It is possible that excess liver Cu decreased hepatic glucose production, contributing the decreased serum glucose concentrations in HIGH, but this cannot be attributed directly to liver damage based on the data collected.

Steers with excess liver Cu had greater expression of *NRF2* in cells recovered from BAL on day 7, indicating a cellular response to oxidative stress. Taken into consideration with the increased clinical scores and ablated nutritional immunity response in the HIGH treatment, this could indicate that excess Cu heightened the severity of respiratory disease, leading to more oxidative stress in the respiratory tract which the steers were responding to by increasing antioxidant production via *NRF2* transcription. Perhaps, since signs of clinical disease were more moderate in the ADE treatment, less antioxidant production was needed and less *NRF2* transcription was detected.

It has been well documented that physiological stressors and inflammation can result in decreased tight junction protein integrity and increased gastrointestinal tract permeability (Hietbrink et al., 2009; Crawford et al., 2022). This can allow bacterial components, such as LPS, to enter circulation from the gastrointestinal tract and worsen the disease state (Kvidera et al., 2017). Because excess Cu is a pro-oxidant (Bremner, 1998) and oxidative stress can damage the intestinal barrier, we hypothesized that the HIGH steers might experience greater gastrointestinal tract permeability during the disease challenge. This hypothesis was further supported by the tendency for increased *NRF2* transcription in the cells isolated from BAL samples in the HIGH steers, indicating a greater need for antioxidant mechanisms. To test this, we used Cr–EDTA, a marker that is poorly absorbed across an intact intestinal barrier, with increased plasma Cr concentrations indicating increased permeability (Wood et al., 2015). However, in the present study, no treatment differences in plasma Cr concentrations 4 h after dosage were noted on either day. This may indicate the effect of excess Cu on disease severity was independent of total gastrointestinal tract integrity. It remains possible that a true difference was not detected due to insufficient statistical power, and more targeted research with greater statistical power should be conducted to fully rule out this mechanism.

In conclusion, excess liver Cu resulted in an ablated nutritional immunity response to infection and heightened severity of disease. Although serum haptoglobin was not influenced by treatment, it appears excess liver Cu concentrations could contribute to the poor health of beef-on-dairy calves in feedlots. This work indicates that over-fortifying diets with Cu may have negative impacts on the health of beef-on-dairy calves.

## Abbreviations:

ADE: adequate liver Cu treatment
ALP: alkaline phosphatase
BAL: bronchoalveolar lavage
BRD: bovine respiratory disease
BRSV: bovine respiratory syncytial virus
BW: body weight
CFU/mL: colony-forming units per milliliter
Cr–EDTA: chromium–EDTA
Cu: copper
ELISA: enzyme-linked immunosorbent assay
FRAP: ferric reducing antioxidant power
ICP–OES: inductively coupled plasma–optical emission spectrometry
LPS: lipopolysaccharide
TMR: total mixed ration
TUS: thoracic ultrasonography
Zn: zinc
